# Complete genome sequence of a *Kayfunavirus* bacteriophage identified in Blantyre, Malawi

**DOI:** 10.1128/mra.00867-25

**Published:** 2025-11-10

**Authors:** Chimwemwe Mhango, Wanangwa Ndovie, Allan Zuza, End Chinyama, Flywell Kawonga, Ernest Matambo, Benjamin Kumwenda, Arox W. Kamng'ona, Chrispin Chaguza, Martin M. Nyaga, Celeste M. Donato, Khuzwayo C. Jere

**Affiliations:** 1 Biomedical Sciences Department, School of Life Sciences and Allied Health Professions, Kamuzu University of Health Sciences 37610, Blantyre, Malawi; 2 Veterinary and Ecological Sciences, Institute of Infection, University of Liverpool 4591, Liverpool, United Kingdom; 3 Malawi-Liverpool-Wellcome Programme, Blantyre, Malawi; 4 Doctoral School of Exact and Natural Sciences, Jagiellonian University 37799, Krakow, Poland; 5 University of St Andrews 7486, Scotland, United Kingdom; 6 Pharmacy Department, School of Life Sciences and Allied Health Professions, Kamuzu University of Health Sciences, Blantyre, Malawi; 7 Department of Host-Microbe Interactions, St. Jude Children's Research Hospital 5417, Memphis, Tennessee, USA; 8 Next Generation Sequencing Unit and Division of Virology, Faculty of Health Sciences, University of Free State 108140 , Bloemfontein, South Africa; 9 Enteric Diseases Group, Murdoch Children’s Research Institute, Parkville, Melbourne, Australia; 10 Department of Medical Laboratory Sciences, Kamuzu University of Health Sciences, School of Life Sciences and Allied Health Professions 37610, Blantyre, Malawi; Portland State University, Portland, Oregon, USA

**Keywords:** *Kayfunavirus*, bacteriophage, DNA sequencing, DNA assembly

## Abstract

We report a bacteriophage from Malawi recovered during rotavirus RNA sequencing. It shares 84.3% nucleotide identity with MH400309, a *Kayfunavirus* bacteriophage. This incidental finding shows RNA virus sequencing can also detect bacteriophages, offering insights into the broader viral diversity in clinical and environmental samples.

## ANNOUNCEMENT

Gastroenteritis remains a significant public health challenge, especially in children under 5 years of age living in low-income settings, and is caused by a range of pathogens, including viruses, bacteria, and parasites. Rotavirus is the leading cause of severe viral gastroenteritis worldwide, though co-infections with bacterial pathogens such as *Escherichia coli* are common and may complicate clinical outcomes (1, 2). The gut microbiome’s viral components, including bacteriophages (viruses that specifically infect bacteria), play essential roles in shaping bacterial population dynamics, influencing bacterial behavior, and potentially modulating host-pathogen interactions (3–5). Here, we report the complete genome sequence of a *Kayfunavirus* bacteriophage assembled during the *de novo* assembly of rotavirus genomes from a stool sample.

A stool sample was collected on 24 October 2019 from a child presenting with acute gastroenteritis at the Queen Elizabeth Central Hospital, Blantyre, Malawi. We screened the stool sample for rotavirus using an enzyme immunoassay (Rotaclone; Meridian Bioscience, Cincinnati, OH, USA). Nucleic acid was then extracted from the rotavirus-positive stool sample using the Trizol-chloroform method for double-strand ribonucleic acid (dsRNA) (6, 7). We enriched for dsRNA using lithium chloride precipitation (8). We further used DNase I (Sigma-Aldrich, Dorset, UK) to remove contaminating DNA (7) before cDNA synthesis (6). We used the cDNA to prepare sequencing libraries using the Nextera DNA Flex Library Preparation Kit (Illumina, San Diego, CA, USA) and sequenced them on an Illumina MiSeq platform with a V3 reagent kit, as previously described (8, 9). A total of 417,142 raw paired-end reads were generated from the MiSeq run.

For the data analysis, all tools were run with default parameters unless otherwise specified. Raw sequence reads were assessed for quality using multiQC v.1.21 (10) and trimmed to remove low-quality bases and adapters with Trimmomatic v.0.39 (11). After quality control, 408,696 paired-end reads were retained and assembled *de novo* using metaSPAdes v.3.15.5 (12). The resulting assemblies were annotated using Genome Detective (13), revealing a 39,229 bp contig with hallmark bacteriophage genomic features. Completeness and contamination of the contig was evaluated using checkV v.0.7.0 (14) (Table 1). To validate the discovery, we reassembled the raw reads using a different short-read metagenomic pipeline on the CZ-ID platform (15).

**TABLE 1:** Summary of the complete bacteriophage genome, gene prediction, and life cycle characteristics

Sample_id	CHX131
NCBI accession	PV611674.1
Contigs	1
Assembly length (bp)	39,229 bp
Number of mapped reads	38,884
Source	*Homo sapiens*
Assembly method	*De novo*
Average sequencing depth	96.5×
Sequencing platform	Illumina Miseq
Read type	Paired end
Average read length (bp)	137.5
G + C content (%)	49.96
No. of genes	50
Viral genes	45
No. of annotated CDS	44
Completeness (%)	100
Contamination (%)	0
Life cycle	Virulent
Genus	*Kayfunavirus*

Open reading frames were predicted using Prodigal with the meta option v.2.6.3 (16), resulting in the identification of 50 genes, of which 45 were specific to viral functions. The predicted genes were annotated using Bakta v.2.14.1 (17). Additionally, Life Cycle Classifier v.1.6.0 in phageAI (18) was employed to assign the phage’s lifestyle (Table 1). Comparative analyses using taxMyPhage v.0.3.5 (19) revealed an average nucleotide identity of 83.9% between the assembled genome and MH400309, a *Kayfunavirus* genus.

The discovery of this bacteriophage highlights the hidden diversity of organisms within the gut virome and underscores the importance of using unbiased assembly pipelines to explore viral diversity. Future work will focus on recovering this bacteriophage from the fecal material and characterizing its biological properties.

## Data Availability

The complete genome of this bacteriophage has been deposited at GenBank under the accession number in Table 1. The raw sequencing reads generated for this study have been deposited in the National Center for Biotechnology Information Sequence Read Archive under accession number SRX30043303 and BioProject accession number PRJNA1303374.
